# Intrapericardial localization of solitary fibrous tumour: a case report

**DOI:** 10.1093/ehjcr/ytaf422

**Published:** 2025-08-26

**Authors:** José Martín Alanís-Naranjo, Omar Alejandro Gil-Guzmán, Daniel Velasco-Ortiz, Ma Delia Pérez Montiel-Gómez

**Affiliations:** Cardiovascular Imaging Department, Instituto Nacional de Cardiología Ignacio Chávez, Juan Badiano 1, Mexico City 14080, Mexico; Cardiology Department, Hospital Regional 1° de Octubre, Instituto de Seguridad y Servicios Sociales de los Trabajadores del Estado (ISSSTE), Instituto Politécnico Nacional 1669, Mexico City 07760, Mexico; Cardiovascular Surgery Department, Hospital Regional 1° de Octubre, Instituto de Seguridad y Servicios Sociales de los Trabajadores del Estado (ISSSTE), Instituto Politécnico Nacional 1669, Mexico City 07760, Mexico; Pathology Department, Instituto Nacional de Cancerología, San Fernando 22, Mexico City 14080, Mexico

**Keywords:** Case report, Cardiac tumours, Cardiovascular imaging, Solitary fibrous tumour

## Abstract

**Background:**

A solitary fibrous tumour (SFT) is a rare fibroblastic tumour, with primary cardiac SFT extremely rare. We report a rare case of a patient presenting with sudden dyspnoea who was diagnosed with a primary cardiac SFT.

**Case summary:**

A 56-year-old woman with a history of diabetes and active smoking presented with sudden dyspnoea. Computed tomography scan revealed a mass adjacent to the left ventricle. In addition to the large pericardial effusion, no signs of cardiac tamponade or valvulopathies were found on echocardiography. The patient underwent open-heart surgery and mass removal, finding a tumour located inside the pericardial sac and attached to the left ventricle’s lateral wall; it did not invade other heart structures. Histological and immunohistochemical examination of the mass revealed SFT diagnosis. The patient was discharged from the hospital in full health, and follow-up examinations revealed no evidence of tumour recurrence.

**Discussion:**

Solitary fibrous tumour most commonly occurs in middle-aged patients and is not gender specific. Multimodal imaging is crucial for diagnosing and managing SFT. A definitive diagnosis must be based on both immunohistochemical and histopathological findings. STAT6 immunoexpression is the most reliable marker for histopathology diagnosis. Given the high SFT recurrence rate, follow-up is essential.

Learning points

- Solitary fibrous tumour is a rare fibroblastic tumour, with intrapericardial localization even rarer.
- Multimodal imaging is essential for diagnosing and managing cardiac solitary fibrous tumours.
- The definitive diagnosis of a solitary fibrous tumour is based on histopathological and immunohistochemical findings, with STAT6 immunoexpression being the most reliable marker.
- Solitary fibrous tumours have a high recurrence rate, which makes long-term follow-up essential.

## Introduction

Solitary fibrous tumour (SFT) is a rare, anatomically ubiquitous fibroblastic tumour, most often found in deep somatic soft tissue and body cavities (especially the pleura, pelvis, and retroperitoneum).^1,2^ Cases of cardiac SFT are exceedingly rare in the literature.^2,3^

Herein, we report the case of a patient who presented with sudden dyspnoea and was diagnosed with a primary cardiac SFT.

## Summary figure

**Figure:**
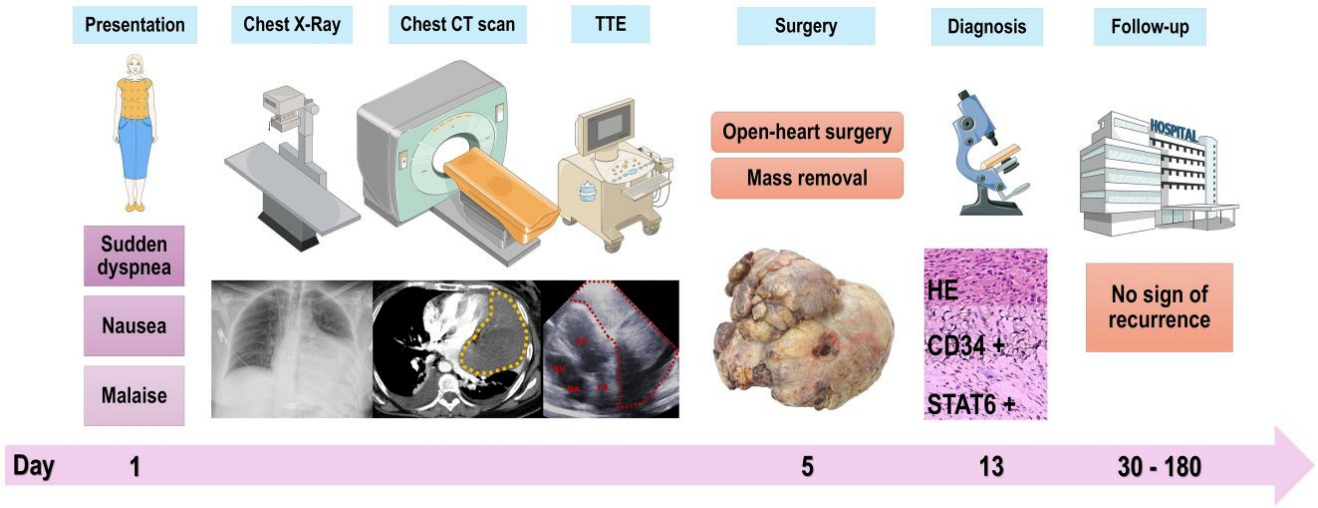


## Case report

A 56-year-old woman presented to the hospital due to sudden dyspnoea, nausea, and malaise. In addition to Type 2 diabetes and active smoking (an average of one cigarette per day for 30 years), the patient denied having ever been diagnosed with any heart disease.

The vital signs at admission were only remarkable for low SaO₂ (88% in ambient air) and tachypnoea (23 breaths per minute); heart rate (75 beats per minute), blood pressure (112/68 mmHg), and temperature (36.3°C) were unremarkable. On physical examination, the patient had audible heart sounds without heart murmurs or jugular ingurgitation but had diminished tactile fremitus on palpation, diminished breath sounds at auscultation, and stony dull percussion notes on the posterior bilateral pulmonary basal fields.

**Figure 1:**
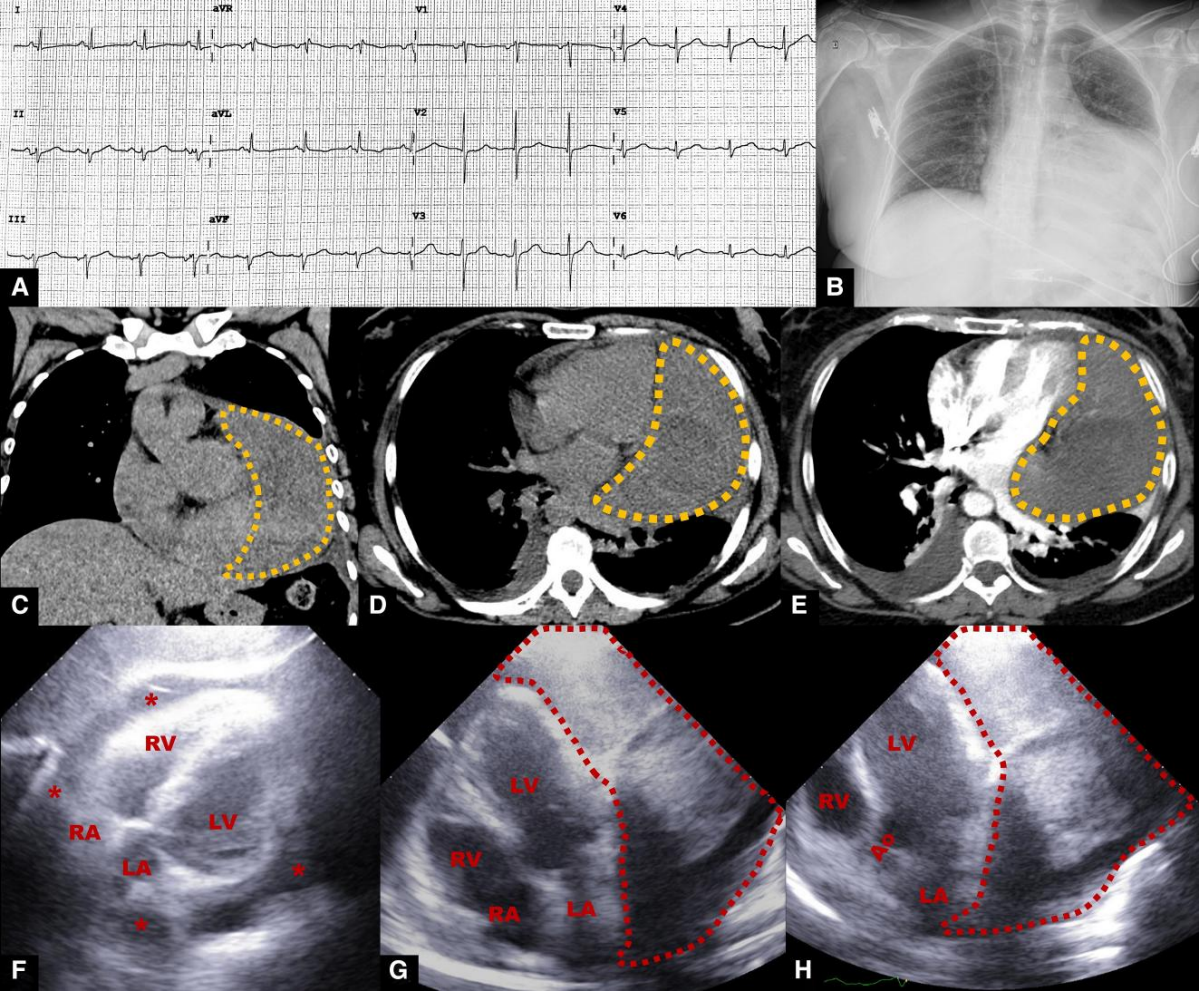
Initial diagnostic approach of the cardiac tumour. (*A*) Twelve-lead electrocardiogram: sinus rhythm with left anterior fascicular block. (*B*) Chest X-ray: pulmonary venous hypertension with cardiomegaly due to enlarged left ventricle. (*C–E*) Contrast chest computed tomography scan: bilateral pleural effusion with mass adjacent to the left ventricle (dotted line mark). (*F–H*) Transthoracic echocardiogram: large pericardial effusion (asterisk) with a homogeneous mass adjacent to the left ventricle (dotted line mark). (*F*) Subcostal four-chamber view, (*G*) apical four-chamber, and (*H*) apical five-chamber view. Ao, aortic valve; LA, left atrium; LV, left ventricle; RA, right atrium; RV, right ventricle.

The electrocardiogram revealed sinus rhythm with a left anterior fascicular block (*Figure 1A*), and the chest X-ray showed pulmonary venous hypertension and an enlarged left ventricle (*Figure 1B*). Only moderately high levels of alanine transaminase were relevant in blood tests (77 U/L, normal range: 10–42 U/L).

Contrast chest computed tomography (CT) scan ruled out pulmonary embolism but showed bilateral pleural effusion and a mass in the mediastinum dependent on the pericardium adjacent to the left ventricle (*Figure 1C–E*). Transthoracic echocardiogram (TTE) revealed a large pericardial effusion and a homogeneous mass adjacent to the left ventricle; no tamponade signs or valvulopathies were detected, and the left ventricular ejection fraction was 73% (*Figure 1F–H*; Supplementary material online, *Video S1*).

According to the Heart Team’s decision, the patient underwent emergency open-heart surgery and mass removal. During the intraoperative period, 270 mL of blood was drained from the pericardial sac. The tumour was located inside the pericardial sac and attached to the left ventricle’s lateral wall; it did not invade other heart structures.

**Figure 2:**
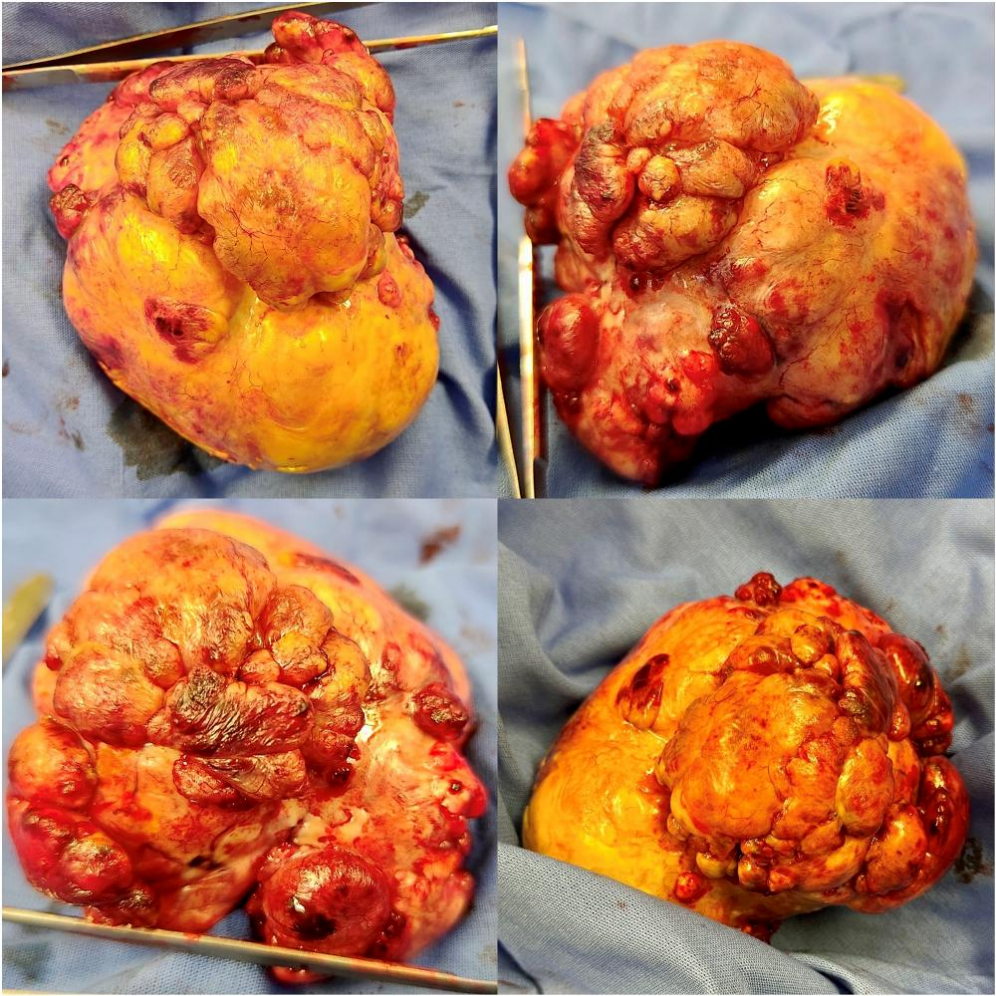
Intraoperative images of solitary fibrous tumour.

**Figure 3:**
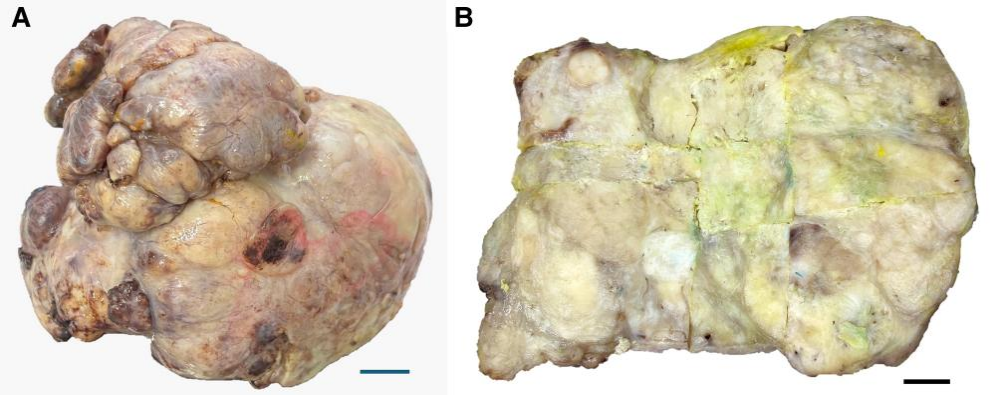
Cardiac solitary fibrous tumour. (*A*) Gross specimen: nodular tumour with a multilobed surface of firm consistency, in brown and grey colours weighing 586 g and measuring 11 × 11 × 10 cm. (*B*) Cut sections: tumour of firm consistency, with a lobed surface and smooth internal lining. A thin, fibrous-looking capsule delimits the tumour.

**Figure 4:**
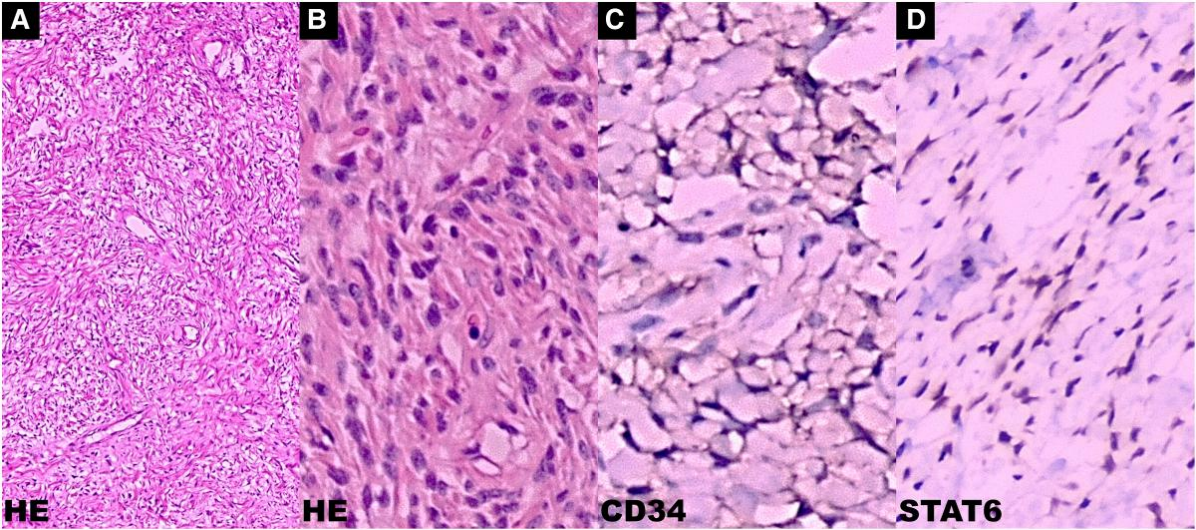
Cardiac solitary fibrous tumour: histological evaluation. Haematoxylin and eosin (*A* and *B*): spindle-shaped cells with ‘pattern-less pattern’, cells without atypia with thin-walled blood vessels alternating with areas of cells without atypia, showing an apparent vascular pattern and collagen in cords. In the immunohistochemical test, the cells were positive for CD34 (*C*) and STAT6 (*D*). HE, haematoxylin and eosin.

The tumour was resected entirely (*Figure 2*), and the specimen was sent for histopathological examination. The intraoperative and postoperative courses were uneventful. Following histological and immunohistochemical examinations, the mass was diagnosed as SFT (*Figures 3* and *4*); a mitotic count of 1 per 10 high-power fields, high cellularity, no necrosis, and no cellular pleomorphism were also noted. The case was concluded as primary cardiac SFT following further imaging studies conducted during hospitalization that were non-diagnostic for SFT metastasis.

The patient was discharged in good general health, and follow-up evaluations showed no evidence of tumour recurrence on echocardiography and chest X-rays.

## Discussion

Solitary fibrous tumour is a soft tissue tumour originating from mesenchymal tissue.^4^ Patients with SFTs are usually in their middle age with no gender-specific differences in incidence.^2^ The tumour size varies greatly depending on its location, with a median tumour size between 7 and 10 cm.^2,4^

Cardiac SFTs frequently manifest no symptoms, cause no pain, grow gradually, and are usually benign.^3^ Symptoms may depend on the extent and anatomic location of the tumour, ranging from complaints including exertional and resting dyspnoea to, or less commonly, fatigue, chest discomfort, dry cough, palpitations, syncope, or generalized oedema.^2,3^

In addition to compressing the cardiac chambers and lungs, giant cardiac tumours can cause heart failure and acute pulmonary hypertension due to obstruction of circulation.^5^ Giant intrapericardial tumours cause dyspnoea by reducing lung volume and elevation of ventricular filling pressures.^6^

Multimodal imaging is crucial for diagnosing and managing a cardiac SFT.^7^ As a first-line imaging modality, chest X-ray typically shows marked cardiomegaly.^2^ Besides detecting cardiac mass and pericardial effusion, echocardiography can also reveal small intratumoral calcifications.^2,4^

Cardiac magnetic resonance (CMR) findings in SFT usually include intermediate signal intensity on T1-weighted images and variable signal intensity on T2-weighted images, well-defined borders, high vascularity, and ‘map’-like inhomogeneous enhancement.^2,7^ Although CMR patterns in SFT are variable, they may be difficult to distinguish from malignant tumours, such as angiosarcomas.^7^

The definitive diagnosis is based on both histopathological findings and immunohistochemical staining.^4^ Histopathology demonstrates a broad spectrum of findings, including haphazardly arranged spindled to ovoid cells with branching, thin-walled staghorn-shaped blood vessels, and prominent stromal collagen.^1^

Bcl2, CD34, and CD99 immunohistochemical staining are commonly used in SFT but are variable in intensity and distribution.^3,8,9^ The positivity ranges for CD99, CD34, and Bcl2 in SFT cases are 57.1%–80%, 80%–92.9%, and 70%–86%, respectively.^8,9^

Nevertheless, expression has been observed in other types of tumours; CD34 expression can be detected in other fibroblastic and myofibroblastic tumours but is lost in aggressive SFTs, whereas BCL-2 is expressed in other fibroblastic spindle cell sarcomas. Furthermore, CD99 expression is observed in early stages or benign tumours, but absent or reduced in advanced stages or malignant tumours.^9^

The diffuse nuclear immunoreactivity for STAT6 by immunohistochemistry is consistent with this tumour type’s underlying NAB2–STAT6 fusion characteristic.^1^ STAT6 immunoexpression on tumour cells can distinguish SFT from its histological mimics.^3^ Additional TERT promoter and TP53 alterations have been associated with aggressive behaviour and dedifferentiation.^1^

Even though there are no specific guidelines for treating SFTs, surgical excision is the method of choice.^3^ Despite the benign nature of most SFTs, ∼5%–10% of them will recur or metastasize, often to the lungs, liver, or bone.^1^ For this reason, it is crucial to obtain adequate negative margins as it decreases the rates of metastases and local recurrence.^2,3^

Relapses are more likely to occur within the first 5 years of follow-up, and rare recurrences are reported within the next 10 years. Five-year survival probability varies between 59% and 100%, whereas 10-year survival varies between 73% and 89%.^3^

The association between histology and the clinical course of the disease is highly unpredictable.^1,3^ According to the 2020 WHO Classification of Tumours of Soft Tissue and Bone, risk stratification models are preferred over the traditional benign/malignant distinction based on microscopic findings in patients with SFT.^1^ Risk-prognostic models have been established in the literature that provide accurate prognoses and risk stratification of SFTs^10–12^ (see Supplementary material online, *Tables S1*–*S3*).

According to the Demicco system,^10^ our patient fulfilled the moderate risk category with a metastasis-free rate of 90% at 5 and 10 years. Concerning the Pasquali system,^12^ our patient fulfilled the low-risk category with a disease-free survival time of 88.9% at 5 years. Based on Salas’ personalized calculator,^11^ our patient has a 9.3% and 15% risk of local recurrence at 5 and 10 years, respectively; the patient’s metastatic recurrence incidence is 11.7% and 18.8% at 5 and 10 years, respectively.

Despite the excellent short- and long-term surgical outcomes of left-sided benign primary cardiac tumour resections, with most patients considered cured after the procedure,^13^ there is no standardized follow-up surveillance programme for cardiac SFT, nor one based on recurrence or metastatic risk. A routine surveillance protocol of 1–2 years with TTE and 3–5 years with CMR or cardiac CT could be recommended for SFT patients after surgery. Suspicion of SFT recurrence or clinical status changes will require immediate multimodal imaging evaluation.

The patient described in this case report was a previously asymptomatic middle-aged woman with a sudden onset of dyspnoea whose multimodal imaging revealed a cardiac mass. STAT6 staining confirmed SFT diagnosis. Based on the risk models previously described,^10–12^ our patient was considered to have a low-intermediate risk of recurrence or metastasis despite the successful surgical excision of the tumour; therefore, close follow-up was necessary. The Heart Team decided to perform surveillance in the first and sixth months, followed by a TTE annual follow-up. Fortunately, there has been no evidence of recurrence on follow-up echocardiograms and chest X-rays.

## Conclusion

Cardiac SFT is a rare and challenging disease to diagnose and treat. The case report highlights the importance of considering unusual diagnoses when working up with cardiac masses. STAT6 is the most reliable histopathological marker for SFT diagnosis. In this setting, multimodal imaging protocols are essential to improve diagnostic accuracy and guide surgical procedures. Additionally, long-term follow-up is imperative due to SFT’s high recurrence rate. Further studies are needed to strengthen the utility of SFT risk-prognostic models.

## Lead author biography


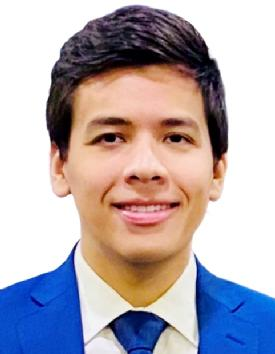


Martín Alanís-Naranjo completed his medical training at the Michoacán University of San Nicolás de Hidalgo in Morelia, Mexico. He subsequently undertook a residency programme in Internal Medicine and Cardiology at the National Autonomous University of Mexico. Currently, he serves as a fellow in cardiovascular imaging at the Instituto Nacional de Cardiología Ignacio Chávez in Mexico City. Cardiac multimodal imaging is among his main interests in cardiovascular medicine.

## Supplementary Material

ytaf422_Supplementary_Data

## Data Availability

The data underlying this article are available in the article and in its online Supplementary material.

## Notes

**Consent:** The authors confirm that written consent for submission and publication of this case report including images and associated text has been obtained from the patient in accordance with the COPE guidelines.

**Funding:** None declared.
