# Supplementary material for: Intrapericardial localization of solitary fibrous tumour: a case report
Source: Eur Heart J Case Rep. 2025 Aug 26;9(9):ytaf422. doi: 10.1093/ehjcr/ytaf422 (PMC12418934; doi:10.1093/ehjcr/ytaf422)
Supplement: ytaf422_Supplementary_Data [file ytaf422_supplementary_data.zip › Supplementary Files.docx]

**Supplementary Files**

**Video S1:** Transthoracic echocardiographic imaging of large pericardial effusion with a mass adjacent to the left ventricle.

**Table S1.- Demicco Risk Stratification System**

**Table S2. Salas Recurrence and Metastasis Risk Model**

**Table S3.- Pasquali Recurrence Risk Model**
