# Supplementary material for: Intrapericardial localization of solitary fibrous tumour: a case report
Source: Eur Heart J Case Rep. 2025 Aug 26;9(9):ytaf422. doi: 10.1093/ehjcr/ytaf422 (PMC12418934; doi:10.1093/ehjcr/ytaf422)
Supplement: ytaf422_Supplementary_Data [file ytaf422_supplementary_data.zip › Tables.docx]

**TABLES**

| **Table S1. Demicco Risk Stratification System** | | | |
| --- | --- | --- | --- |
| **Risk factor** | **Score** | **Risk factor** | **Score** |
| **Age**  <55  ≥55 | 0  1 | **Tumor necrosis**  < 10%  ≥ 10 % | 0  1 |
| **Tumor size (cm)**  < 5  5 to <10  10 to <15  ≥ 15 | 0  1  2  3 | **Mitotic count**  **(x 10 high-power fields)**  0  1-3  ≥ 4 | 0  1  2 |
| **Risk class** | **Total score** | **Metastasis-free** | |
|  |  | **5 -years** | **10-years** |
| Low  Intermediate  High | 0-3  4-5  6-7 | 100%  90%  27% | 100%  90%  - |
| Adapted from *Demicco EG, Wagner MJ, Maki RG, Gupta V, Iofin I, Lazar AJ, et al. Risk assessment in solitary fibrous tumors: validation and refinement of a risk stratification model. Mod Pathol. 2017 Oct;30(10):1433-1442.*^10^ | | | |

| **Table S2. Salas Recurrence and Metastasis Risk Model** | | | |
| --- | --- | --- | --- |
| **Local recurrence** | | **Metastatic recurrence** | |
| **Risk factor** | **Score** | **Risk factor** | **Score** |
| **Age** | Years | **Age** | Years |
| **Localization**  Viscera  Soft tissue | 1  0 | **Localization**  Limb  Others | 0  1 |
| **Radiotherapy**  Yes  No | 0  1 | **Mitotic count (x 10 high-power fields)**  ≤4  >4 | 0  1 |
| Adapted from *Salas S, Resseguier N, Blay JY, Le Cesne A, Italiano A, Chevreau C, et al. Prediction of local and metastatic recurrence in solitary fibrous tumor: construction of a risk calculator in a multicenter cohort from the French Sarcoma Group (FSG) database. Ann Oncol. 2017 Aug 1;28(8):1979-1987.*^11^ | | | |

| **Table S3. Pasquali Recurrence Risk Model** | | | | |
| --- | --- | --- | --- | --- |
| **Variables** | **Score** | | | |
| **Mitotic Rate**  Low  High >4 (x 10 high-power fields) | **0**  **3** | | | |
| **Cellularity**  Low  Moderate-High | **0**  **2** | | | |
| **Pleomorphism**  Low  Moderate-High | **0**  **2** | | | |
| **Risk**  Very low  Low  Intermediate  High | **Total score**  **0**  **2**  **3-5**  **>5** | | | |
| **Disease-free survival time** | **Very low risk** | **Low risk** | **Intermediate risk** | **High risk** |
| 1-year | 100 | 96.6 | 94.4 | 78.5 |
| 3-year | 100 | 92.8 | 82 | 52.6 |
| 5-year | 100 | 88.9 | 65.9 | 52.6 |
| Adapted from *Pasquali S, Gronchi A, Strauss D, Bonvalot S, Jeys L, Stacchiotti S, et al. Resectable extra-pleural and extra-meningeal solitary fibrous tumours: A multi-centre prognostic study. Eur J Surg Oncol. 2016 Jul;42(7):1064-70.*^12^ | | | | |
